# So-called “American Sheep Disease”

**Published:** 1895-06

**Authors:** 


English veterinarians should be extremely careful in not making an error in regard to their so-termed "American Sheep Disease." Their blunder on contagious pleuro-pneumonia has already placed them in an unenviable light among American veterinarians. They should remember that tuberculosis is an extremely rare disease in the sheep family; that diseases of parasitic origin are quite common in sheep the world over. The disease, so far as we glean from English veterinary journals, is of a parasitic character, is not new, does not confine itself to America, and as a rule is only discovered post-mortem or when being killed in the abbatoirs for consumption, so that as a rule it can be said to cause little or no appreciable disturbance in the animal's health.

